# ASHA-Led Community-Based Groups to Support Control of Hypertension in Rural India Are Feasible and Potentially Scalable

**DOI:** 10.3389/fmed.2021.771822

**Published:** 2021-11-22

**Authors:** Michaela A. Riddell, G. K. Mini, Rohina Joshi, Amanda G. Thrift, Rama K. Guggilla, Roger G. Evans, Kavumpurathu R. Thankappan, Kate Chalmers, Clara K. Chow, Ajay S. Mahal, Kartik Kalyanram, Kamakshi Kartik, Oduru Suresh, Nihal Thomas, Pallab K. Maulik, Velandai K. Srikanth, Simin Arabshahi, Ravi P. Varma, Fabrizio D'Esposito, Brian Oldenburg

**Affiliations:** ^1^Department of Medicine, School of Clinical Sciences at Monash Health, Monash University, Melbourne, VIC, Australia; ^2^Kirby Institute, University of New South Wales, Sydney, NSW, Australia; ^3^Achutha Menon Centre for Health Science Studies, Sree Chitra Tirunal Institute for Medical Sciences and Technology, Trivandrum, India; ^4^Global Institute of Public Health, Ananthapuri Hospitals and Research Institute, Trivandrum, India; ^5^George Institute for Global Health, University of New South Wales, Sydney, NSW, Australia; ^6^George Institute for Global Health, New Delhi, India; ^7^School of Population Health, University of New South Wales, Sydney, NSW, Australia; ^8^Department of Population Medicine and Lifestyle Diseases Prevention, Medical University of Białystok, Białystok, Poland; ^9^Cardiovascular Disease Program, Department of Physiology, Biomedicine Discovery Institute, Monash University, Melbourne, VIC, Australia; ^10^Department of Public Health & Community Medicine, Central University of Kerala, Kasaragod, India; ^11^Melbourne School of Population and Global Health, University of Melbourne, Melbourne, VIC, Australia; ^12^Westmead Applied Research Centre, University of Sydney, Sydney, NSW, Australia; ^13^Department of Cardiology, Westmead Hospital, Sydney, NSW, Australia; ^14^School of Public Health and Preventative Medicine, Monash University, Melbourne, VIC, Australia; ^15^Melbourne School of Population and Global Health, Nossal Institute for Global Health, University of Melbourne, Melbourne, VIC, Australia; ^16^Rishi Valley Rural Health Centre, Chittoor, India; ^17^Department of Endocrinology, Diabetes and Metabolism, Christian Medical College, Vellore, India; ^18^Peninsula Clinical School, Central Clinical School, Monash University, Frankston, VIC, Australia; ^19^Baker Heart and Diabetes Institute and Larobe University, Melbourne, VIC, Australia

**Keywords:** hypertension control, self-management, community-based, task-shifting, implementation evaluation, accredited social health activist, rural, India

## Abstract

**Background:** To improve the control of hypertension in low- and middle-income countries, we trialed a community-based group program co-designed with local policy makers to fit within the framework of India's health system. Trained accredited social health activists (ASHAs), delivered the program, in three economically and developmentally diverse settings in rural India. We evaluated the program's implementation and scalability.

**Methods:** Our mixed methods process evaluation was guided by the United Kingdom Medical Research Council guidelines for complex interventions. Meeting attendance reports, as well as blood pressure and weight measures of attendees and adherence to meeting content and use of meeting tools were used to evaluate the implementation process. Thematic analysis of separate focus group discussions with participants and ASHAs as well as meeting reports and participant evaluation were used to investigate the mechanisms of impact.

**Results:** Fifteen ASHAs led 32 community-based groups in three rural settings in the states of Kerala and Andhra Pradesh, Southern India. Overall, the fidelity of intervention delivery was high. Six meetings were delivered over a 3-month period to each of the intervention groups. The mean number of meetings attended by participants at each site varied significantly, with participants in Rishi Valley attending fewer meetings [mean (SD) = 2.83 (1.68)] than participants in West Godavari (Tukeys test, *p* = 0.009) and Trivandrum (Tukeys test, *p* < 0.001) and participants in West Godavari [mean (SD) = 3.48 (1.72)] attending significantly fewer meetings than participants in Trivandrum [mean (SD) = 4.29 (1.76), Tukeys test, *p* < 0.001]. Culturally appropriate intervention resources and the training of ASHAs, and supportive supervision of them during the program were critical enablers to program implementation. Although highly motivated during the implementation of the program ASHA reported historical issues with timely remuneration and lack of supportive supervision.

**Conclusions:** Culturally appropriate community-based group programs run by trained and supported ASHAs are a successful and potentially scalable model for improving the control of hypertension in rural India. However, consideration of issues related to unreliable/insufficient remuneration for ASHAs, supportive supervision and their formal role in the wider health workforce in India will be important to address in future program scale up.

**Trial Registration:** Clinical Trial Registry of India [CTRI/2016/02/006678, Registered prospectively].

## Introduction

Hypertension is a major modifiable risk factor for cardiovascular disease. In 2019, high systolic blood pressure (SBP) was the leading global risk factor for attributable death and was responsible for 10–20% of Disability Adjusted Life Years (1).

In India, rural regions, while having similar prevalence of hypertension to urban regions, have poorer awareness and control of hypertension. In their large systematic review, Anchala and colleagues found that the prevalence of hypertension in rural areas was ~25% compared to 33% in urban areas, while awareness was less in rural (25%) than in urban areas (42%) (2). Furthermore, only 10% of the rural population with hypertension and 20% of the urban population with hypertension have their blood pressure (BP) under control (2).

Poor control of hypertension in rural India, similar to findings from elsewhere in the world, may be attributable to poor knowledge and awareness of hypertension (3), as well as a shortage of health care providers, non-availability of medications, and the relative high cost of treatment when treatment is available (4). Poor control may also be affected by physician inertia, especially the lack of knowledge of the latest guidelines for the management of hypertension and the concept of prehypertension (5).

Community health workers (CHWs)/non-physician health workers (NPHWs), lay health workers, lay health advisors, peers, and others may be an important avenue for improving the control of hypertension. Indeed, there have been positive effects of employing these workers/volunteers in assisting and maintaining health behavior changes in programs for maternal and child health, HIV/AIDS, diabetes, and cardiovascular disease (6, 7). Furthermore, in an analysis of task sharing in eight projects funded by the Global Alliance for Chronic Diseases, task-sharing between CHWs/NPHWs and doctors was shown to be feasible and potentially scalable to deliver care in hard-to-reach and poorly resourced settings in low-and middle-income countries (LMICs) (8, 9).

In India, CHWs, called Accredited Social Health Activists (ASHAs), are an important component of the 2005 National Rural Health Mission, to improve access of rural people to effective primary healthcare (10, 11). ASHAs are predominantly female, non-physician, community-based volunteers, whose work complements that of Auxiliary Nurse Midwives (ANMs) and act as a bridge between the community and the healthcare system. ASHA have existing relationships with their communities and thus may be suitably placed to provide a community-based program to rural communities. Accountability for the work of the ASHAs primarily lies within the purview of the Village Health Sanitation and Nutrition Committee which is a committee formed at the revenue village level and acts as a subcommittee of the village council. A 2011 evaluation of the ASHA program identified the challenges of integrating a largely volunteer and incentivized workforce into the country's health and human resource strategy (12).

During 2014–16, we conducted a cluster randomized controlled trial (cRCT) to test the effectiveness of an ASHA-led, community-based group program for improving the control of hypertension in rural India (CHIRI) in three settings, each at a different stage of economic and epidemiological transition (13, 14). Within the three settings, 637 participants from five intervention clusters and 1,097 participants from 10 control clusters were recruited between November 2015 and April 2016, with follow-up occurring in 459 participants in the intervention group and 1,012 participants in the control arm.

We found that reduction in both SBP and diastolic BP (DBP) was more in the intervention group than in the control group in all the three study areas. The proportion of participants with control of hypertension was greater in the intervention group than in the control group in two of the three study sites (13).

We conducted an evaluation of the CHIRI program implementation (intervention arm only) with the aim of determining its fidelity, the barriers to and enablers of the program, possible mechanisms of impact and the potential for future scalability of ASHA-led community-based group programs to improve the control of hypertension in rural India.

## Methods

### Setting and Sample

Details of the protocol and main outcomes have been published earlier (13, 14). Briefly, the CHIRI study was done in two phases, the first of which included (1) a baseline survey to identify people with hypertension; (2) focus group discussions (FGDs) and in-depth interviews (IDIs) to identify individual- and system-level barriers to diagnosis and control of hypertension; and (3) a cross-sectional survey to determine the availability, affordability, and accessibility of medicines essential for treatment of hypertension, type 2 diabetes, and secondary prevention of cardiovascular disease.

Informed by the knowledge and data gained during phase 1, phase 2 tested the feasibility and effectiveness of community-based group meetings to support individuals in the self-management of hypertension. Within the three settings, 637 participants from five intervention clusters and 1,097 participants from 10 control clusters were recruited between November 2015 and April 2016, with follow-up occurring in 459 participants in the intervention group and 1,012 participants in the control arm.

Eligible participants for phase 2 were adult women and men aged at least 18 years with hypertension (defined as SBP ≥ 140 mmHg and/or DBP ≥ 90 mmHg and/or taking anti-hypertensive medication), identified in the phase 1 baseline survey and residing in the study settings randomized to receive the intervention.

### Intervention Theory

The intervention was based on the CHW peer support model described by Heisler (15). The principles of the Chronic Disease Self-Management Program and behavior change guided the mechanism of impact (16). Program content, known to significantly contribute to improved self-management, included knowledge, and understanding of the disease, promotion of uptake of healthy behavior and clinical interaction through goal setting and active engagement in monitoring and treatment (7, 15, 17).

### Implementation Framework

**Figure 1:**
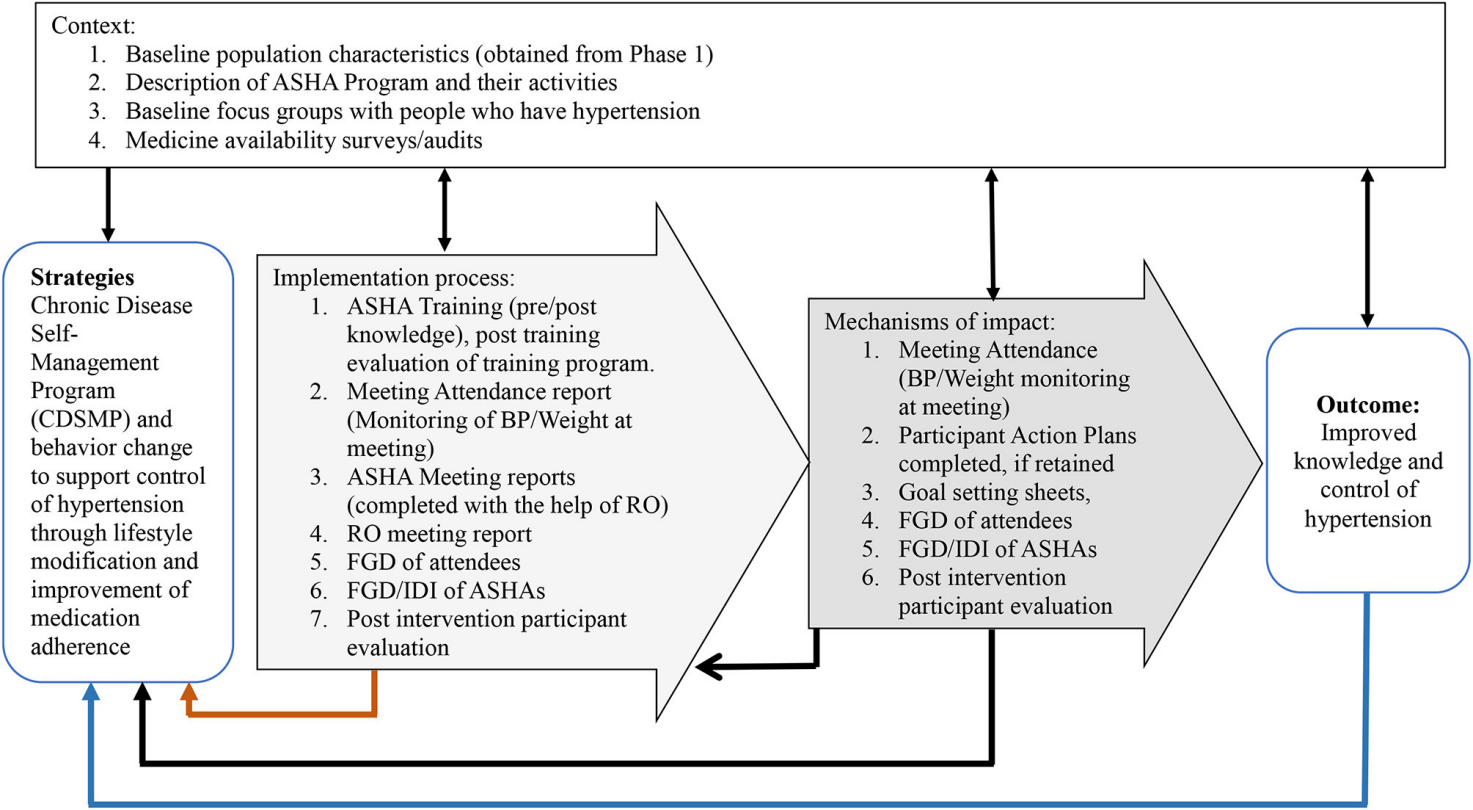
Description of intervention activities/data tools to inform process evaluation using the United Kingdom Medical Research Council framework (17). ASHA, Accredited Social Health Activist; BP, Blood Pressure; RO, Research Officer; FGD, Focus Group Discussion; IDI, In Depth Interviews.

We used the Intervention Evaluation Process Model developed by the United Kingdom Medical Research Council guidelines for complex interventions, which focuses on three components: context, implementation process, and mechanisms of impact (see Figure 1) (18). Phase 1 of the study was used to understand the context and to inform the development of the intervention with respect to the prevalence of hypertension, current awareness of hypertension, knowledge about hypertension and use of health services, and availability of medicines (14).

We used an evaluation of the ASHA training (19) to provide details about the future feasibility of using ASHAs as the workforce to deliver the program. The fidelity of the program and its dose were assessed through (1) meeting reports completed by ASHAs with support from Research Officers (ROs), during and after the community meetings; (2) monitoring and evaluation sheets completed by the ROs, and; (3) meeting attendance and BP/adherence monitoring sheets that were recorded by the ASHAs in the meeting resources manual (available from Figshare https://figshare.com/s/b94c7af22ae220540c45).

At their final visit, participants provided feedback on the program, including details about the support they received from family and friends, their perception about their relationships with health care providers, and their perceived support from the ASHAs who conducted the meetings. Post-intervention FGDs, held separately with ASHAs and participants, provided additional information regarding feasibility and potential scalability of the intervention. The mechanisms of impact were assessed through the same meeting reports completed by ASHAs and ROs during and after the community meetings, as outlined in point (1) above.

### CHIRI Intervention

Details of the intervention protocol and its impact evaluation have been described previously (13, 14). As outlined (13), during the development of the program we met with stakeholders/policy makers, including experts from the Ministry of Health, Indian Council of Medical Research, and independent researchers to obtain input about how best to tailor our approach to fit within the Indian Health system. ASHAs were financially remunerated to conduct the group meetings in accordance with the existing schedule for Village Health Sanitation and Nutrition Committee Activities (20) and Village Health and Nutrition Days (21). At the local administrative level, the study was conducted with permission from the local community leaders in each region.

#### ASHA Training

We conducted a pilot of the ASHA training program with ASHAs, who were not involved in delivering the program, and local clinicians, which enabled refinement of the training and program resources, as well as the delivery of the training program.

ASHAs were trained over 5 days to lead community groups and support control of hypertension through improving participants' knowledge of hypertension, using goal setting to adopt healthier lifestyles, and monitoring of blood pressure and weight (14, 19).

The ASHAs and ROs were provided with a training manual which included details of each education session, the requirements for working within a research project (particularly with respect to completing reports and meeting details that were used to evaluate the implementation as outlined above), group facilitation skills, ethics, and self-care. ASHA were supported during each meeting by an RO from the study team. The RO was available to assist the ASHA to answer questions and clarify meeting messages if required. At the end of each meeting the ROs reviewed, with ASHAs, the content of the meeting and all activities undertaken in each meeting (available from Figshare https://figshare.com/s/7bbfcc22e0c9c91a5ca0?file=11780321).

#### CHIRI Program

The program consisted of six fortnightly sessions, each of ~90 min, during which ASHAs recorded the attendance of participants, weight and blood pressure, adherence to BP medication, and any visit to a health care provider (HCP) for hypertension-related illness in the previous 2 weeks. The content of the meetings included knowledge of hypertension, encompassing risk factors, control of hypertension, physical activity, diet, practical self-management, and strategies to continue control of hypertension after the final meeting (14). Participants also received handouts to take home to remind them of the ideas and messages discussed at the meetings and to assist them with self-management actions between meetings. For example, at each meeting participants received a pictorial monitoring chart to record foods eaten, exercise undertaken, and salt, tobacco, and alcohol reduction in between meetings.

At the beginning of each meeting, participants were encouraged to share their experiences from the previous 2 weeks, achievements in self-management, and any difficulties encountered. Then, ASHAs delivered the meeting content using flipcharts, provided by the study team. These flipcharts (and handouts) were mostly pictorial and were delivered in the local language (Malayalam in Trivandrum and Telugu in West Godavari and Rishi Valley) (Meeting flip charts and handouts are available at: Figshare https://figshare.com/s/7bbfcc22e0c9c91a5ca0).

SMART (Specific, Measurable, Actionable, Realistic, Time limited) goal setting was practiced during each meeting. Participant goals were recorded by the ASHAs for review at the following meeting. When reviewing goals, during the meetings, participants were encouraged to share difficulties they may have experienced in achieving their set goals and group members were invited to share potential solutions and experiences when facing similar difficulties in achieving goals.

### Data Collection for Evaluation of Implementation

ASHAs were provided with a handbook of meeting resources (Figshare- https://figshare.com/s/b94c7af22ae220540c45), to be used at and after each meeting. Each of the following, which were located within the handbook, were completed by the ASHAs, and subsequently used to assess the fidelity, intervention dose, and adherence to the protocol, as part of this evaluation:

1. The “Meeting Attendance and BP/Adherence Monitoring Sheet” was used to record the meeting date and theme, participant attendance, number of additional support persons/family attendees, BP and heart rate, weight of each participant, each participant's use of medication or visits to an HCP for BP-related issues in the past 2 weeks. This sheet was part of the program as it provided a record of each participant's attendance and previous BP/weight for the ASHA to monitor at each meeting. This sheet also provided a means to assess fidelity to the program, including the dose of intervention received by each participant.
2. The “Goal Setting Sheet” was used to track individual participant's goals set during the meeting to enable review at the next meeting. These sheets comprised part of the program and were reviewed to assess the fidelity to the program and to confirm goal setting activities at each meeting.
3. Post-meeting reports:
  a. “Meeting Report:” This report was completed by ASHAs with support from ROs, during and after the community meetings, and included specific details of the content and management of each meeting, the number of participants and support persons/family attendees and use of supplied resources and support from RO assessed using a 5-point Likert scale “none/little—0–20%” to “a lot of information—81–100%.” Motivation of participants was rated by ASHAs on a 5-point Likert scale from “not at all motivated” to “very motivated.” The following were also collected by the ASHAs: (i) The time taken by ASHAs to prepare for the meeting, (ii) meeting activities, (iii) challenges faced by ASHAs in conducting the meetings, (iv) assessment by ASHAs of the positive and negative aspects of the meetings, (v) any additional activities undertaken in their community related to hypertension and chronic disease management, and time spent on those activities, and (vi) additional engagement by ASHAs with village leaders or other health care professionals.
  b. “Monitoring and Evaluation Sheet for Research Officers:” This sheet was completed by the RO attending the meeting, to provide an objective assessment of the fidelity and dose of the intervention at each meeting. The report included details of the topics covered during the meeting, timing of meeting activities, and a checklist of ASHAs activities during meetings (BP/weight measurement of attending participants, goal revision from previous meeting, use of the flip chart for the meeting, goal setting, use of the action plan tool). Additional space was available to record any issues raised by the participants with respect to (i) barriers or difficulties the participants had in achieving their goals, and (ii) difficulties, barriers and potential solutions raised by the participants during the meeting for controlling hypertension.

At the final assessment, the participants randomized to receive the intervention completed an evaluation survey (see Appendix 1) seeking the participant's perspective of attendance, support, and assistance to manage BP received from the ASHA, HCPs, members of the family, friends/support persons, and other members of the community. This information contributed to our understanding of the mechanisms of impact. A Likert scale was used for some questions in this evaluation survey. Each participant evaluated “how well do you think the ASHA helped you manage your high BP on a day-to-day basis?” using a 3-point Likert scale (“Not at all,” “Some of the time,” “All of the time”). The question about “how useful” various people had been in assisting their management of hypertension was graded on a 7-point Likert scale from “A little useful” through to “Extremely useful.” The question about how much the group meetings helped with day-to-day management of hypertension was measured using a 3-point Likert scale (“Not at all,” “A little bit,” “A great deal”). Another question about “how much support/encouragement did the (various) meeting activities give you to manage your BP on a daily basis?”, was measured on a 4-point Likert scale (“No support,” “Little support,” Moderate support,” “A lot of support,” “Not applicable;” the latter was selected if the participant did not receive or complete the activity).

Focus Group Discussions were conducted with ASHAs after completion of the program. The interview guide is available on Figshare (https://figshare.com/s/b94c7af22ae220540c45). Among those randomized to participate in the program, we conducted FGDs with those who chose to attend the program group meetings (see Appendix 2: Interview guide 1a), and IDIs with those who chose not to attend any of the group meetings (see Appendix 2: Interview guide 1b). These were conducted ~2 months after the program was completed and were done to separately determine the perceptions of these two distinct groups. These discussions were audio-recorded, transcribed verbatim and general themes investigated as described below.

### Data Analysis

Data from the “Meeting Reports” and “Monitoring and Evaluation Sheet for Research Officers” were entered into a Microsoft Access 2007–2016 database and the tabulated data were analyzed using Stata IC/14.0 (StataCorp, College Station TX, USA). Basic descriptive data are presented as means (standard deviation), medians (quartile 1–quartile 3) and number (%). Differences in means between sites were assessed using analysis of variance followed by Tukey's test. For categorical variables, Pearson's χ^2^-test was used, and Bonferroni correction was used to account for the three comparisons. Two-tailed *P* ≤ 0.05 was considered statistically significant.

Qualitative data were analyzed by deductive coding using a simple framework matrix derived from questions/discussion points from the discussion guides. An excel spreadsheet was developed using the questions/discussion points from the discussion guides (see Appendix 2). Codes included motivation to attend meetings, barriers and enablers to meeting attendance, new knowledge gained during the program, use of the resources provided for the program, support from family/friends, other group members and ASHAs, BP monitoring, and access to medication after the meetings ended.

## Results

### Context

Context was assessed using the framework developed by Daivadanam et al. (22). The CHIRI study was conducted in three rural settings in Southern India, each at a different stage of economic and epidemiological transition. The population in the Trivandrum district in the State of Kerala have a higher life expectancy and more than 93% literacy (late transition region). The population in the Rishi Valley (early transition region) in Chittoor District in the southern part of AP consists mainly of subsistence farmers and ~50% have no formal schooling. West Godavari district in coastal Andhra Pradesh (AP) is economically between the two other regions and literacy in 2011 was 74.6% (23). At the individual/family level, in the context of BP, baseline mean SBP was greater in participants in the Rishi Valley than in the other two settings. Mean baseline DBP differed across all settings with the Rishi Valley participants having the highest and participants in Trivandrum the lowest (13). Participants in the Rishi Valley generally were less literate, had lower educational attainment, and a greater proportion were below the poverty line, than those in either West Godavari or Trivandrum. At the community level, 11 of the villages selected for the program had existing ASHAs while four villages did not have ASHAs, so we specifically recruited one woman from each of those four villages to fulfill the role of ASHA for the purposes of this study. Training of the ASHAs successfully led to increased knowledge of hypertension, skills, and motivation which has previously been evaluated and described (19).

At the healthcare setting, access to services was variable, with participants in the Rishi Valley reporting greater difficulty in accessing health care services than those at the other two sites. Seventeen pharmacies (51%; 10 public, 5 private, 2 other) had at least one drug from each class of the recommended cardiovascular disease preventive therapy combination of antiplatelet, statin, ACE/ARB, plus beta blocker or other BP lowering drugs. One private pharmacy did not carry any essential medicines for cardiovascular disease, BP, or type 2 diabetes mellitus, while the remaining 32 (97%) carried at least one glucose lowering agent and one BP lowering agent at the time the survey on the availability of medicines was undertaken.

### Program Fidelity

A total of 15 ASHAs delivered the program in 15 villages. Four ASHAs in Rishi Valley led six community groups, seven ASHAs in West Godavari led 14 community groups and four ASHAs in Trivandrum led 12 community groups. A total of 192 meetings (36 Rishi Valley, 84 Godavari, and 72 Trivandrum) were conducted. Two groups in the Rishi Valley were combined for meetings four to six, but attendance data and other meeting data were analyzed per group. Each group completed six community-based meetings over a 3-month period, and all delivered the meeting content as planned, using the flip charts provided as recorded by the RO in the “Monitoring and Evaluation Sheet for Research Officers.” At the beginning of each group meeting all attending participants had their BP, weight, heart rate, medication adherence and any hypertension-related health care visits since the last meeting recorded by ASHAs into the “Meeting Attendance and BP/Adherence Monitoring Sheet” in the ASHA resource manual. At each of the meetings goal setting was undertaken by the participants and goals were recorded by ASHAs in the “Goal Setting Sheet” for each participant in the ASHA resource manual.

On average, ASHAs reported spending 73.7 (SD 42.2) min preparing for each meeting. ASHAs in West Godavari reported spending significantly more time preparing for meetings [mean 86.7 (SD 42.4) min] than ASHAs in Trivandrum [60.7 (SD 43.7) min; Tukeys test, *p* < 0.001]. Meeting preparation time for ASHAs in Rishi Valley [69.6 (SD 29.0) min] was not significantly different to that for ASHAs in Trivandrum (Tukeys test, *p* = 0.53) or West Godavari (Tukeys test, *p* = 0.09). ASHAs accessed “some,” (defined as 51–80%) or “a lot,” (defined as 81–100%) of information from the training resources during meeting preparation for 181/192 (94.3%) of the meetings and “some” or “a lot” of information from the RO for 181/192 (94.3%) of the meetings. Fidelity in delivering the program in accordance with the protocol was high.

### Program Exposure

A total of 637 participants consented to participate in the community group meetings. The average age of participants was 56.6 years (SD 14.3) and 58.7% were female (13). Overall, 416 (65.3%) of participants attended at least one meeting. The participation rate varied by site with West Godavari having the greatest participation rate and Trivandrum the least (Table 1).

**Table 1:** Meeting attendance by study site.

**Study site**	**Total recruited**	Number participants (% participation^*^)	**Number participants attending per meeting Mean (SD)**	**Number participants attending per meeting Median (IQR)**	**Total meetings attended per participant [Mean (SD)]**	**Total meetings attended per participant Median (IQR)**	**Number (%) attending only 1 meeting**	**Number (%) attending all 6 meetings**	**Community member attendance Mean (SD)**
Rishi Valley	135	103 (76.3)	8.1 (4.2)	7 (5–11)	2.83 (1.68)^A^^‡^, *B*§	3 (1–4)	33 (32)	9 (8.7)	0.28 (0.57)
West Godavari	198	162 (81.8)	6.7 (2.4)^A*C*§^	6 (5–8)	3.48 (1.72)^C§^	4 (2–5)	31 (19.7)	24 (14.8)	3.4 (3.37)^A§*C*§^
Trivandrum	304	151 (49.7)	9.1 (2.3)	9 (7–11)	4.29 (1.76)	5 (3–6)	23 (15.2)	48 (31.8)	1.3 (1.6)
Total	637	416 (65.3)	7.8 (3.0)	7 (5–10)	3.61 (1.81)	4 (2–5)	87 (20.9)	81 (19.5)	

*SD, standard deviation; IQR, interquartile range*.

* *Participation defined as attending at least one community meeting. For continuous variables, if P_Region_ ≤ 0.05, Tukey's test was used to determine which regions differed at P ≤ 0.05. This is shown by superscript (A = RV vs. WG, B = RV vs. T, C = WG vs. T, and P ≤ 0.05*,

‡ *P < 0.01*,

§ *P < 0.001)*.

Overall, approximately eight participants attended each of the meetings. Significantly fewer participants, on average, attended meetings in West Godavari compared with attendance by participants in Rishi Valley (Tukeys test, *p* = 0.04) or Trivandrum (Tukeys test, *p* < 0.001). Overall, each participant attended approximately four meetings. Program exposure based on the mean number of meetings attended by participants at each site varied significantly, with participants in Rishi Valley attending fewer meetings [mean (SD) = 2.83 (1.68)] than participants in West Godavari (Tukeys test, *p* = 0.009) and Trivandrum (Tukeys test, *p* < 0.001) and participants in West Godavari [mean (SD) = 3.48 (1.72)] attending significantly fewer meetings than participants in Trivandrum [mean (SD) = 4.29 (1.76), Tukeys test, *p* < 0.001]. Overall, 87/416 participants (20.9%) attended only one meeting, with the greatest proportion in the Rishi Valley. Approximately one in five (81/416; 19.5%) attended all six meetings, the greatest proportion being in Trivandrum. Lack of time (47.3%) and health issues (17.3%) were the main reasons cited by participants for not attending meetings. Attendance at meetings by a support person of the participant was significantly greater in West Godavari than Rishi Valley (Tukeys test, *p* < 0.001) and Trivandrum (Tukeys test, *p* < 0.001; Table 1), with no detectable difference between Trivandrum and Rishi Valley. According to the protocol, meetings were scheduled to last 90 min, including the time taken to measure BP and weight. Overall, the average duration of meetings was 78.1 min (SD 28.0; Table 2). The meetings were longest in Trivandrum, this being the region with the greatest number of participants attending per session, so having the longest duration for recording of BP.

**Table 2:** Meeting duration by study site.

**Study site**	**Mean (SD) meeting duration minutes**	**Mean (SD) duration for measurement of BP/Weight minutes**
Rishi Valley	61.8 (19.0)	23.8 (9.2)
West Godavari	65.7 (16.4)	17.1 (7.0)^A§*C*§^
Trivandrum	100 (28.3)^B§*C*§^	33.9 (9.4)^B§^
Total	78.1 (28.0)	24.7 (11.3)

*SD Standard deviation, For continuous variables, if P_Region_ ≤ 0.05, Tukey's test was used to determine which regions differed at P ≤ 0.05. This is shown by superscript (A = RV vs. WG, B = RV vs. T, C = WG vs. T, and ^§^P < 0.001)*.

### Mechanisms of Impact

Motivation of participants was rated by ASHAs as “very motivated” for 89.1% (171/192) and “motivated” for the remaining 10.9% (21/192) of meetings. ASHAs in West Godavari tended to rate participants as “very motivated” (96.4%) more often than ASHAs from Rishi Valley (80.6%) or Trivandrum (84.7%) (*p* = 0.04, Pearson χ^2^ with Bonferroni correction).

To understand possible mechanisms of impact of the program outside of the meeting sessions, ASHAs were asked to report any additional support they provided to participants outside the meetings as well as any additional information they provided to their communities, other than program participants, about hypertension, other chronic diseases, or information about the CHIRI program. ASHAs in West Godavari reported providing additional support to participants, in the 2 weeks between every meeting (Table 3). ASHAs provided between-meeting support to participants after most of the group meetings in Trivandrum (69/72) and after each group meeting in West Godavari (84/84) while ASHAs in Rishi Valley provided between-meeting support after three group meetings (3/36). ASHAs in West Godavari were in general significantly more likely to provide between-meeting support to participants than ASHAs in Rishi Valley, although all ASHAs provided some support to participants between meetings for management of hypertension, medication adherence, alcohol cessation/reduction and assistance with visiting HCPs. ASHAs in West Godavari and Trivandrum discussed aspects of the program with community leaders or health service providers while ASHAs in Rishi Valley did not report any discussions with community leaders or health services staff (data not shown).

**Table 3:** Mean duration (minutes) of between-meeting support given by ASHAs to participants.

**Type of support**	**Rishi Valley**	**West Godavari**	**Trivandrum**	**Total**	***p*** **-value**
	**Mean duration in minutes (SD)** **(Min–Max)**	**Mean duration in minutes (SD)** **(Min–Max)**	**Mean duration in minutes (SD) (Min–Max)**	**Mean duration in minutes (SD)** **(Min–Max)**	
Hypertension management	0.28 (1.7) (0–10)	13.6 (9.8) (0–60)	13.3 (11.65) (0–60)	11.0 (10.9) (0–60)	<0.001^A, B^
Goal setting and review	0	10.2 (5.1) (0–30)	8.1 (8.3) (0–30)	7.5 (7.2) (0–30)	<0.001^A, B^
Medication adherence strategies	1.8 (10.0) (0–60)	8.2 (5.1) (0–20)	4.8 (6.1) (0–20)	5.7 (7.0) (0–60)	<0.001^A^ 0.006 ^C^
Tobacco cessation	0	9.6 (4.7) (0–30)	1.3 (4.4) (0–30)	4.7 (6.0) (0–30)	<0.001^A, C^
Alcohol cessation/reduction	0.14 (0.8) (0–5)	8.8 (7.5) (0–30)	1.4 (3.8) (0–20)	4.4 (6.7) (0–30)	<0.001^A, C^
Need for clinical advice	0	8.4 (6.2) (0–20)	4.8 (8.3) (0–40)	6.8 (7.4) (0–40)	<0.001^A^ 0.001 ^B^ 0.002 ^C^
BP monitoring	0	10.3 (7.8) (0–30)	9.5 (14.7) (0–90)	8.1 (11.1) (0–90)	<0.001^A, B^
Assistance with visiting health care provider/facility	2.5 (11.1) (0–60)	5.1 (9.0) (0–60)	1.9 (6.6) (0–50)	3.4 (8.7) (0–60)	>0.05^A, B^, = 0.05^C^
Assistance with family negotiation	0	8.3 (7.0) (0–30)	0.4 (1.8) (0–10)	4.7 (6.6) (0–30)	<0.001^A, C^

*ASHAs, Accredited Social Health Activist; SD, Standard deviation; BP, Blood Pressure; Min, Minimum; Max, Maximum. For continuous variables, if P_Region_ ≤ 0.05, Tukey's test was used to determine which regions differed at P ≤ 0.05. This is shown by superscript (A = RV vs. WG, B = RV vs. T, C = WG vs. T)*.

Nearly three quarters (467/637, 73.3%) of participants invited to participate in the group meetings responded to the participant evaluation survey. Three hundred and fifty-six of those responding (76.2%) attended at least one meeting and provided a response about attending the meetings with a support person or family member. Of these, 79 (22.2%) attended with a support person (Table 4). Support from family and friends to implement information gained at the meetings to improve blood pressure control was reported “very often” by ~45.8% (163/356) of participants. Participants reported receiving support “very often” more frequently in Rishi Valley (50.9%) and Trivandrum (63.5%) than West Godavari (27.3%). Overall, 85.0% of participants reported that attending group meetings did not change their relationship with their HCP. A greater proportion in Rishi Valley stated an unchanged relationship with their HCP (96.5 vs. 81.8% in West Godavari, 83.5% in Trivandrum), but these apparent differences by site were not statistically significant.

**Table 4:** Number of participants who attended group meetings with a support person and assessment of the frequency of support, received by participants to use meeting information to improve BP, from support person who attended meetings with the participant and from family/friends who did not attend the group meetings, by study site.

**Study site**	**Number of participants who attended meeting with support person *n* (%/site)**	**Frequency of between meeting support received from support person** ***n*** **(%)**			**Frequency of between meeting support received from family/friends** ***n*** **(%)** ^‡^		
		**Not often**	**Sometimes**	**Very often**	**Not often**	**Sometimes**	**Very often**
Rishi Valley (*n* = 57)^*^	10 (17.5)	0	5 (50.0)	5 (50.0)	7 (12.3)	21 (36.8)	29 (50.9)
West Godavari (*n* = 154)^*^	43 (27.9)	9 (20.9)	13 (30.2)	21 (48.8)	65 (42.2)	47 (30.5)	42 (27.3)
Trivandrum (*n* = 145)^*^	26 (17.9)	0	8 (32.0)	17 (68.0)	12 (8.3)	41 (28.3)	92 (63.5)
Total (*N* = 356)^*^	79 (22.2)	9 (11.5)	26 (33.3)	43 (55.1)	84 (23.6)	109 (30.6)	163 (45.8)

*BP, Blood Pressure*,

* *111 missing data (12 Rishi Valley, 22 West Godavari, 77 Trivandrum). Pearson χ^2^ with Bonferroni correction for multiple comparisons (3 regions) p = 0.23*,

‡ *Pearson χ^2^ with Bonferroni correction for multiple comparisons (3 regions), p = 0.003*.

Generally, participants in West Godavari rated the extent of help and support received from ASHAs in daily management of high BP more supportively than participants in Trivandrum and Rishi Valley, particularly with respect to behaviors relating to diet and medications (Table 5).

**Table 5:** Participant evaluation of help from ASHAs during the group meetings to manage high BP on a day-to-day basis.

**To what extent did the ASHA**	Rishi Valley^*^ ***n*** **= 57**			**West Godavari** ***n*** **= 154**			**Trivandrum**^‡^ ***n*** **= 145**			**Total** ***N*** **= 356**		
	**Not at all**	**Some of the time**	**All of the time**	**Not at all**	**Some of the time**	**All of the time**	**Not at all**	**Some of the time**	**All of the time**	**Not at all**	**Some of the time**	**All of the time**
	***n*** **(%)**	***n*** **(%)**	***n*** **(%)**	***n*** **(%)**	***n*** **(%)**	***n*** **(%)**	***n*** **(%)**	***n*** **(%)**	***n*** **(%)**	***n*** **(%)**	***n*** **(%)**	***n*** **(%)**
Help you to remember to take your medication	54 (94.7)	3 (5.3)	0 (0)	6 (3.9)	85 (55.2)	63 (40.9)	109 (75.2)	36 (24.8)	0 (0)	169 (47.5)	124 (34.8)	63 (17.7)
Help you to get your medications	56 (98.3)	1 (1.8)	0 (0)	15 (9.7)	85 (55.2)	54 (35.1)	110 (75.9)	35 (24.1)	0 (0)	181 (50.8)	121 (34.0)	54 (15.2)
Ask you about problems with medications/effects	57 (100)	0 (0)	0 (0)	80 (52.0)	63 (40.9)	11 (7.1)	116 (80.0)	29 (20.0)	0 (0)	253 (71.1)	92 (25.8)	11 (3.1)
Help you with monitoring your BP	57 (100)	0 (0)	0 (0)	12 (7.8)	84 (54.6)	58 (37.7)	104 (71.7)	41 (28.3)	0 (0)	173 (48.6)	125 (35.1)	58 (16.3)
Remind you to see HCP regularly even when well	56 (98.3)	1 (1.8)	0 (0)	60 (39.0)	60 (39.0)	34 (22.1)	129 (89.0)	16 (11.0)	0 (0)	245 (68.8)	77 (21.6)	34 (9.6)
Help you to build better communication with HCP	57 (100)	0 (0)	0 (0)	84 (54.6)	59 (38.3)	11 (7.1)	128 (88.3)	17 (11.7)	0 (0)	269 (75.6)	76 (21.4)	11 (3.1)
Remind and help you to put your needs first	56 (98.3)	1 (1.8)	0 (0)	89 (58.6)	53 (34.9)	10 (6.6)	119 (82.1)	26 (17.9)	0 (0)	264 (74.6)	80 (22.6)	10 (2.8)
Reminding and helping you to eat more fruit/veg	55 (96.5)	2 (3.5)	0 (0)	5 (3.3)	44 (28.6)	105 (68.2)	128 (88.3)	17 (11.7)	0 (0)	188 (52.8)	63 (17.7)	105 (29.5)
Remind and help you to reduce portion size	56 (98.3)	1 (1.8)	0 (0)	11 (7.1)	70 (45.5)	73 (47.4)	125 (86.8)	19 (13.2)	0 (0)	192 (54.1)	90 (25.4)	73 (20.6)
Remind and help you to do 30 min. daily physical activity	56 (98.3)	1 (1.8)	0 (0)	5 (3.3)	61 (39.6)	88 (57.1)	119 (82.1)	26 (17.9)	0 (0)	180 (50.6)	88 (24.7)	88 (24.7)
Remind and help you to reduce oily foods/salt/sugar in your diet	56 (98.3)	1 (1.8)	0 (0)	1 (0.7)	48 (31.2)	105 (68.2)	112 (77.2)	32 (22.1)	1 (0.7)	169 (47.5)	81 (22.8)	106 (29.8)

*ASHAs, Accredited Social Health Activist; BP, Blood pressure; HCP, Health care practitioner*,

* *12 missing data, 22 missing data*,

‡ *77 missing data*.

Overall, 57.1% of participants thought that the community group meetings helped “A great deal” to manage their high BP on a day-to-day basis. Fewer participants (43.9%) in Rishi Valley reported group meetings helped “A great deal” than in West Godavari (54.6%) and Trivandrum (65.1%) (Pearson χ^2^ with Bonferroni correction, *p* < 0.001). More than two thirds of participants in Rishi Valley (71.9%) and West Godavari (68.0%) believed other factors, in addition to the group meetings, were helpful to them for day-to-day management of BP while in Trivandrum less than three percent of participants stated other factors were helpful (Pearson χ^2^ with Bonferroni correction, *p* < 0.001). Other factors, including reduction of salt in their diet and taking medicine as directed, were identified by participants of Rishi Valley and West Godavari as additionally being of major importance for managing their BP, despite these two messages being part of the group meeting content (Table 6). Participants in West Godavari reported that family support was important for monitoring BP, while participants in Rishi Valley reported that frequent monitoring of BP was helpful.

**Table 6:** Factors/activities other than the group meetings that may have helped participants manage BP on a day-to-day basis.

**Other factors assisting day to day management of high BP**	**Rishi Valley** ***n*** **= 69 (%)**	**West Godavari** ***n*** **= 176 (%)**	**Trivandrum** ***n*** **= 222 (%)**	**Total** ***n*** **= 467 (%)**
Reducing salt in diet	41 (59.4)	76 (43.2)	3 (1.4)	120 (25.7)
Family support	18 (26.1)	89 (50.6)	2 (0.9)	109 (23.3)
More frequent BP monitoring	36 (52.2)	48 (27.3)	2 (0.9)	86 (18.4)
Taking medicine as directed by HCP	19 (27.5)	53 (30.1)	1 (0.5)	73 (15.6)
Information from data collector	0	69 (39.2)	0	69 (14.8)
Increasing green vegetables	15 (21.7)	46 (26.1)	1 (0.5)	62 (13.2)
Support from HCP	3 (4.4)	31 (17.6)	1 (0.5)	35 (7.5)
Regular exercise	3 (4.4)	14 (8.0)	1 (0.5)	18 (3.9)
Attend 104 mobile clinic	0	8 (4.6)	0	8 (1.7)
Other community members	0	5 (2.8)	0	5 (1.1)

#### Focus Group Discussions

Nine FGDs were conducted with participants who attended the group meetings. In Trivandrum, two FGDs were held with 15 participants in each of them. In West Godavari, six meetings were held with an average of nine participants per meeting. In Rishi Valley one FGD was done with six participants. Overall, ~54% of participants attending FGDs were female, and age of participants ranged from 25 to 78 years. Focus group discussions reinforced the findings of the post-program participant evaluation surveys (see Supplementary Table 1). The overwhelming motivation for participants to attend the meetings was to obtain information about hypertension and how to manage it. Participants stated the information about how to control hypertension that they received from their HCPs was basic and consisted mostly of prescriptions for medication and basic dietary advice about salt reduction.

Participants stated the meetings provided them with knowledge about hypertension and how to improve their management of it, by adopting simple and practical lifestyle changes with respect to diet (e.g., demonstration of portion size), exercise (e.g., suggestion to get off the bus one stop earlier than their destination bus stop) and adherence to medication (e.g., using simple medication reminders and monitoring sheets).

Program resources were helpful and were still being used by participants after cessation of the program. The pictorial exercise handout was frequently mentioned as a resource still being used. Participants stated the pictures in the resources were well-understood by educated and illiterate participants alike.

Participants reported that family support was important for ongoing BP control and that dietary changes, such as reduction of salt during meal preparation, was the most frequent form of support.

Not all participants agreed that ASHAs or other group members provided support after the program had ceased, although those who lived near other participants or ASHAs in the villages reported that ASHAs and group members maintained contact with them and enquired about their BP.

Simple goals such as taking tablets as directed may be sustainable over time, but participants expressed some lack of motivation or accountability if their goals were not being reviewed or monitored regularly. Participants in FGD found it useful to get their BP checked at each meeting and felt accomplished when efforts were successful. However, ongoing access to BP monitoring after the program ceased was difficult and participants reported checking their BP only if they felt unwell.

Lack of medication distribution during the meetings was a prominent discussion point by the participants and by the ASHAs. Participants had some expectations that medications might be distributed and, at each FGD, participants and ASHAs expressed views that provision of medications would increase attendance at the meetings. ASHAs reported that lack of provision of medications was a frequent complaint from the participants. Cost of medications was seen as a potential barrier to continued control of BP. Participants considered the BP medication available through the mobile 104 service (mobile services providing scheduled primary health care services to villages of India) to be sub-standard or different from those prescribed by their HCP, driving participants to purchase medications from private suppliers.

Suggestions, from the participants who attended meetings, to improve the program included provision of medications during the meetings, inclusion of free blood sugar monitoring and medication (for diabetes) in addition to BP monitoring, home visits, electronic reminders to attend meetings, assistance with transportation to meetings, ensuring convenient meeting locations and meeting schedules for all residents.

In FGDs conducted with ASHAs after the program was completed, ASHAs expressed views supporting the meetings and the need for their continuation in their communities. Inclusion of past participants, provision of medications and incorporation of home visits were suggested as improvements to future meetings.

“*If we include our participants who are self-managing their BP after participating in our program in the next program and make them share their experience, then it would be useful.”—ASHA West Godavari*

“*Visit people's homes and check their BP at least once in a month. If we do like that, they will concentrate on diet control and exercise.”—ASHA Trivandrum*

“*They will definitely come if we give them injections or tablets.”—ASHA Rishi Valley*

Twelve IDIs in West Godavari and two FGD in Trivandrum were conducted with invitees to the program who declined to attend any group meetings. Those who did not attend the group meetings reported work commitments (at home, agriculture, or workplace), physical disabilities, and carer responsibilities as barriers to attendance. Group meeting participants expressed their willingness to share the knowledge gained in the meetings with their family and community members. Those who did not attend the meetings reported that those who had attended the group meetings had shared information from the group meetings with them.

## Discussion

This process evaluation has shown that the CHIRI study was implemented with good fidelity to all the components of the CHIRI program, as outlined in the original study protocol (14). The ASHA-led training component of the program was implemented with high fidelity (19). All six group meetings were delivered using the program materials (flip charts and meeting handouts) at each study site and during each meeting, and all participants were weighed and had their BP measured at each meeting. Participants attended a median of four of the six meetings and the meetings lasted an average of 78 min. However, there was some variation across study sites. Response rates and participation in the program varied across study sites and the percentage of eligible participants who participated in at least one of these sessions, and who attended all six meetings, varied across study sites. Some of this variation might be explained by the differences in educational attainment, literacy, and main employment types in each setting. Support provided to participants by the ASHAs between meetings, to help manage high BP on a day-to-day basis, varied between study sites as did support received by family and friends of the participants. Even though there were some variations in the implementation of the program model in each of the three CHIRI sites, the effect size and primary outcome (control of hypertension) did not vary significantly across the three sites (13).

ASHAs utilized the training, program resources and support from ROs nearly 95% of the time to prepare for the meetings. During the post program FGDs, ASHAs reported that they felt empowered and motivated to perform the tasks they were trained to do.

Qualitative data obtained from FGDs and IDIs with participants and ASHAs may enhance understanding of the factors that influence the causal relationship between implementation and outcome in a real-world setting (24). Focus Group Discussions helped to understand community perspectives on health care seeking behaviors in general and regarding hypertension and were useful to understand the individual- and system-level barriers to control of hypertension in the three settings.

Dietary changes, specifically salt reduction, and frequent blood pressure monitoring were seen as beneficial by participants in this study. These aspects of the program are evidence-based components of improving control of hypertension (25, 26).

Incorporating support from family and friends into future iterations of the program, by supporting flexibility and willingness of supporters to adapt, may enhance outcomes (27) and was acknowledged as important by participants in this study.

In India, ASHAs are already very important within the National Health Mission (10). Indeed, input from ASHAs and from local stakeholders, including health care professionals and policy makers, contributed to the design and delivery of a program which has a good fit with India's health system. ASHAs and other CHWs have great potential to contribute to community-based health programs in LMIC and their involvement in such programs has been expanding recently in many countries such as Ethiopia, Bangladesh, and India (28). Patient screening, lifestyle education, support for self- management and assistance to navigate the health care system are tasks which can and have been successfully undertaken by CHWs for those with non-communicable diseases (9, 29).

There have been many reports of the use of CHWs in delivering reproductive, maternal, and child health services (30) and cardiovascular risk factor management programs (31–33). These studies identify the importance of structured training, an emphasis on participant education and lifestyle change, cultural adaptation, and resources to support the program (34). In line with this evidence base, the CHIRI study included a 5-day training program, which emphasized enhancing knowledge, culturally appropriate and practical self-management skills, frequent weight, and BP monitoring with automatic BP machines, and a training manual and program resources to support implementation of the program. The program also included ongoing mentoring and support of ASHAs by the research team.

ASHAs identified that the training they had received increased their skills and standing in their communities and within the health workforce. Ongoing supervision and support by the RO during the meetings were also identified as helpful during the program delivery. During the evaluation of the ASHA training program ASHA reported historical issues with timely remuneration and lack of supportive supervision (19). Motivating ASHAs by training them effectively, providing salaries on time and maintaining quality through monitoring and supportive supervision are essential steps in task-sharing (31). Supportive supervision and positive feedback can enable and strengthen the collaboration between the health system, CHWs and their communities. It can also facilitate appropriate data collection to allow for ongoing monitoring and evaluation of a program (35).

When assessing scalability, we know that diagnosis and treatment of hypertension in India is a significant problem (36). Many of the major components of the CHIRI program are transferable for the prevention and self-management of other chronic diseases such as diabetes, cardiovascular disease, stroke, and promotion of good mental health.

The fidelity of and the extent to which the CHIRI intervention was implemented in the three different study sites supports the wider implementation of this approach. The group-based approach of the program is also less resource intensive than an individually delivered approach. The effectiveness of group-based programs for the self-management and prevention of chronic diseases is also supported by several earlier studies (37, 38). Therefore, our approach is very appropriate for a resource-constrained setting.

Interviews with ASHAs in the CHIRI program (19) and other studies (39, 40) identify that current remuneration for ASHAs is insufficient and should be revised in the context of their expanding role in the health system (12, 41). The current position of ASHAs in the wider health workforce in India is an important consideration for future program scalability (42).

### Strengths and Limitations

A major strength of this process evaluation was the inclusion of multiple sources of information collected at several time points in each of the study sites. The inclusion of process measures from the start of the intervention allowed measurement of implementation and process measures over the duration of the study. The presence of the RO at each meeting provided an independent assessment of the implementation process. Some of the measures are based on self-perception and evaluation which may have changed over time between the end of the intervention and the time of evaluation, potentially resulting in recall bias.

Staffing and conducting the community-based group meetings within the context of the existing health workforce in rural India was also a significant strength of the model. All project materials were piloted and refined incorporating local input which enhanced local acceptability and cultural suitability of the program.

Generalizability of this program with respect to urban and other settings in India is a limitation of this study. The sample size of the intervention study was small and restricted to five rural clusters in southern India. Furthermore, attendance at every community meeting by a supervisor or ASHA supporter (like the RO as part of the study team in this study) may not be practical in a real-world setting and other options to provide supportive supervision may need to be explored. Upscaling of this program will require further testing in other states of India which have more diversity in culture and educational attainment.

## Conclusion

The CHIRI program is a good “fit” with the existing health care system of India to address the increasing burden of hypertension as well as other chronic conditions. The program was delivered by ASHAs who have been identified as being very important contributors to the future delivery of such programs in rural India, in particular. The intervention was co-designed with end users, including health care professionals and policymakers. Tools to measure evaluation of the implementation included meeting reports from ASHAs, RO reports that summarized activities occurring in each session, IDIs with health workers, FGDs with participants and IDIs with non-participants, to understand why the project was working or facing difficulties. This process evaluation suggests that the CHIRI intervention and program delivery model is a successful, scalable model based on the outcomes and effectiveness of the program. The capacity of ASHAs to deliver the intervention suggests the future sustainability and scalability of CHIRI delivery as they are already part of the existing rural health services.

## Data Availability Statement

The raw data supporting the conclusions of this article will be made available by the authors, upon reasonable request.

## Ethics Statement

Ethics approval was obtained from Sree Chitra Tirunal Institute for Medical Sciences and Technology (Trivandrum, India; SCT/IEC-484/July-2013), the Centre for Chronic Disease Control (CCDC-IEC-09-2012), Christian Medical College (Vellore, India), the Health Ministry Screening Committee of the Government of India (58/4/1F/CHR/2013/NCD II), and Monash University (CF13/2516–2013001327). All procedures followed were in accordance with the ethical standards of the responsible committee on human experimentation (institutional and national) and with the Helsinki Declaration of 1975, as revised in 2000. Informed consent was obtained from all participants for being included in the study. The patients/participants provided their written informed consent to participate in this study.

## Author Contributions

MR, RJ, KT, CC, BO, RE, AM, KKal, KKar, NT, PM, VS, SA, RV, and AT: conceptualization. MR and GM: formal analysis. MR, GM, and KC: writing—original draft. MR, RJ, KT, CC, BO, RE, AM, KKal, KKar, OS, NT, GM, PM, VS, SA, KC, RV, RG, FD'E, and AT: writing—review and editing. All authors contributed to the article and approved the submitted version.

## Funding

This study was funded by a research grant from the National Health and Medical Research Council (Australia) under the auspices of the Global Alliance for Chronic Diseases (GACD; GNT1040030) Hypertension Research Programme. RJ acknowledges the support of a National Heart Foundation Future Leader Fellowship (102059) and UNSW Scientia Fellowship. AT acknowledges support from the NHMRC for a research fellowship (GNT1042600). PM was supported by UKRI/MRC Grant MR/S023224/1—Adolescents' Resilience and Treatment nEeds for Mental health in Indian Slums (ARTEMIS), and NHMRC/GACD Grant APP1143911—Systematic Medical Appraisal, Referral and Treatment for Common Mental Disorders in India—(SMART) Mental Health. RG is currently supported by a Marie Sklodowska-Curie Actions Research Fellowship (EU Grant Agreement ID 754432) outside this study.

## Conflict of Interest

RG is a shareholder in several global medical and bio-pharmaceutical companies as part of his investment portfolio. The remaining authors declare that the research was conducted in the absence of any commercial or financial relationships that could be construed as a potential conflict of interest.

## Publisher's Note

All claims expressed in this article are solely those of the authors and do not necessarily represent those of their affiliated organizations, or those of the publisher, the editors and the reviewers. Any product that may be evaluated in this article, or claim that may be made by its manufacturer, is not guaranteed or endorsed by the publisher.

## GLOSSARY

**104 Mobile Clinic**-Mobile medical vans that provide primary health care services on fixed days of a week in villages of India. **ASHA-**Accredited Social Health Activist receive 4–5 weeks training, promote reproductive, maternal, neonatal and child health, encouraging immunization, institutional-based deliveries, and family planning and providing home-based newborn care and some medicines, such as oral contraceptives. They also receive incentives for malaria case identification, TB treatment support and conducting village health committee meetings (43). **ANM**-Auxiliary Nurse Midwife, receive 2 years training and provide immunization, health education, antenatal care and conduct deliveries (43).

**AWW-**Angawadi worker, receive 3–4 weeks training, run preschool programs, provide supplementary food to young children, adolescent girls, and lactating women, and provide health and nutrition education to pregnant women, mothers, and adolescent girls (43). **BP-**Blood Pressure. **CHW**-Community Health Worker. **CHIRI-**Controlling Hypertension in Rural India. **FGD-**Focus Group Discussion. **HCP**- Health Care Provider. **IDI-**In-depth Interview. **LMIC-**Low-and Middle-Income Country. **NCD-**Non-Communicable Disease. **NPHW**-Non-Physician Health Worker. **RO**-Research Officer.
