# Supplementary material for: ASHA-Led Community-Based Groups to Support Control of Hypertension in Rural India Are Feasible and Potentially Scalable
Source: Front Med (Lausanne). 2021 Nov 22;8:771822. doi: 10.3389/fmed.2021.771822 (PMC8645590; doi:10.3389/fmed.2021.771822)
Supplement: Supplementary file 1 [file Data_Sheet_1.PDF]

Draft

To help us improve and allow us to expand this program to other rural villages we would like to know your thoughts about the group meetings and the ASHA in helping you manage your high blood pressure.

**ATT1a**	Did you attend all the community group meetings?	Yes ☐ No ☐<br>if **YES** go to **ATT2a**
**ATT1b**	If you did not attend all the community group meetings<br>How many meetings did you attend? if "0" answer **ATT1c, PEV4**	0 ☐ 1 ☐ 2 ☐<br>3 ☐ 4 ☐ 5 ☐
**ATT1c**	Please indicate which factors may have limited your attendance at the group meetings<br>***Cross all that apply***	Health Issues ☐<br>Lack of time ☐<br>Meeting time inconvenient ☐<br>Difficult getting to venue ☐<br>Health Care Provider not supportive ☐<br>Meeting not interesting ☐<br>Meeting too long ☐<br>Meeting too short ☐<br>Meeting not well organised ☐<br>Meeting too frequent ☐<br>Meeting not helpful ☐<br>Other participants ☐<br>Other (please specify below) ☐<br>☐☐☐☐☐☐☐☐☐☐☐☐☐☐☐
**ATT2a**	Did any members of your family or other support person attend any meetings with you?	Yes ☐ No ☐<br>if NO go to **ATT3**
**ATT2b**	How often did your family or support person help you and support you at home or work to use information from the meetings to improve your blood pressure (eg by helping you reduce your salt , increase your vegetables and fruit, be active every day)?	Not often ☐<br>Sometimes ☐<br>Very Often ☐
**ATT3**	How often did your family or friends **who did not attend the meetings** help you and support you at home or work to **use information you learned from the meetings to improve your blood pressure** (eg by helping you reduce your salt , by supporting you to eat more vegetables and fruit, by supporting your effort to be more active every day)?	Not often ☐<br>Sometimes ☐<br>Very Often ☐
**ATT4a**	Has your relationship/the way you communicate with your health care provider changed since you've been attending the group meetings (e.g. do you ask more questions, ask for explanations about your medications, ask about your test results, discuss your difficulties etc)?	Yes ☐ No ☐<br>if Yes go to **ATT4b**
**ATT4b**	**You answered YES to the question above**<br>How has your experience with your health care provider changed?<br>(eg do you ask more questions, ask for explanations about your medications, ask about your test results, discuss your difficulties etc)?	☐☐☐☐☐☐☐☐☐☐☐☐☐☐☐<br>☐☐☐☐☐☐☐☐☐☐☐☐☐☐☐

Draft

The following section asks about your experience of the community group meetings. If your community was offered group meetings please answer the following questions even if you did not attend all of the meetings.

Your answers are anonymous and will not be seen by the ASHA or other community members

PEV 1 How well you think **the ASHA** helped you manage your high blood pressure on a day to day basis?

**PEV 1a**	To what extent did the ASHA help you remember to **take your medication** as your health care provider instructed? (give you some ways to remember to take your medication, help remind you)	Not at all ☐ Some of the time ☐ All of the time ☐
**PEV 1b**	To what extent has the ASHA help you **to get your medications** (by telling you where to go to get them, advising you when the 104 service was coming) ?	Not at all ☐ Some of the time ☐ All of the time ☐
**PEV 1c**	To what extent has the ASHA asked **you about any problems with your medicines or their effects**?	Not at all ☐ Some of the time ☐ All of the time ☐
**PEV 1d**	To what extent did the ASHA help you with **monitoring your blood pressure** (by reminding you to get your blood pressure measured, advising you when the 104 service was coming, asking you about your blood pressure and when you last measured it) ?	Not at all ☐ Some of the time ☐ All of the time ☐
**PEV 1e**	To what extent did the ASHA **remind you to see your health care provider regularly even when you are not sick?**	Not at all ☐ Some of the time ☐ All of the time ☐
**PEV 1f**	To what extent did the ASHA helped you build **better communication skills** to use during your health care visits?	Not at all ☐ Some of the time ☐ All of the time ☐
**PEV 1g**	To what extent did the ASHA remind and help you to **put your needs first** when thinking about managing your blood pressure on a daily basis?	Not at all ☐ Some of the time ☐ All of the time ☐
**PEV 1h**	To what extent didthe ASHA remind and help you to **eat more fresh fruits and vegetables?** (take you shopping, give you recipes, fruit or vegetables from gardens)	Not at all ☐ Some of the time ☐ All of the time ☐
**PEV 1j**	To what extent didthe ASHA **remind and help you to reduce the portions of food** you were eating?	Not at all ☐ Some of the time ☐ All of the time ☐
**PEV 1k**	To what extent did the ASHA or other members of your community group **remind and help you to do 30 minutes of activity each day** (ask you about your exercise, offer to exercise with you, accompany you to group exercise)	Not at all ☐ Some of the time ☐ All of the time ☐
**PEV 1L**	To what extent did the ASHA remind and help you to **reduce oily foods or salt and sugar in your diet**	Not at all ☐ Some of the time ☐ All of the time ☐
**PEV 2**	How much do you think the group meetings in your community have helped you manage your high blood pressure on a day to day basis?	Not at all ☐ A little bit ☐ A great deal ☐

**PEV3 a**	Were there other factors/activities (other than the group meetings) that may have helped you to manage your high blood pressure on a day to day basis?	☐ Yes ☐ No ☐ Don't know<br>If No go to **PEV4**

PEV3 b

Please indicate which factors/activities other than the group meetings may have helped you manage your blood pressure on a daily basis

Cross all that apply

Health Care Provider support ☐	Other community members ☐
Information from data collector ☐	Reducing Salt in diet ☐
More frequent BP monitoring ☐	Increasing green vegetables ☐
Family support ☐	Regular exercise ☐
Taking medicine as told ☐	Attend Mobile 104 ☐
Other (please specify below) ☐	

☐☐☐☐☐☐☐☐☐☐☐☐☐☐☐

PEV 4 Please select those people from whom you get help and advice to manage your blood pressure on a daily basis and for each person you select indicate how useful they have been

	**A little Useful**			**Useful**			**Extremely useful**
ASHA ☐	☐	☐	☐	☐	☐	☐	☐
Group Members ☐	☐	☐	☐	☐	☐	☐	☐
Family/Friends ☐	☐	☐	☐	☐	☐	☐	☐
Health Care Provider ☐	☐	☐	☐	☐	☐	☐	☐
Pharmacist ☐	☐	☐	☐	☐	☐	☐	☐
Other Community Members ☐	☐	☐	☐	☐	☐	☐	☐
No one useful ☐							

PEV 5 How much support or encouragement did the following meeting activities give you tomanage your blood pressure on a daily basis (choose N/A if you did not receive or participate in this activity)

		**No Support**	**Little Support**	**Moderate Support**	**A lot of Support**	**Not Applicable**
**PEV 5a**	Attending group meetings	☐	☐	☐	☐	☐
**PEV 5b**	Goal setting in the group meetings	☐	☐	☐	☐	☐
**PEV 5c**	Problem Solving in the group meetings	☐	☐	☐	☐	☐
**PEV 5d**	Community activity (eg walking group)	☐	☐	☐	☐	☐
**PEV 5e**	Group activities (eg activity class in group)	☐	☐	☐	☐	☐
**PEV 5f**	Visit from health expert to group meeting	☐	☐	☐	☐	☐

Appendix 2: Focus Group Discussion Guide 1a
Hypertensives Focus Group Discussion Guide – for those who did attend
Questions:

1. You recently attended the group meetings on high blood pressure. What was your motivation for attending these meetings?
2. What enabled you to attend these meetings?
3. Did you attend all of the meetings?
    a. If not, what were the main reasons for not attending the meetings? Explore barriers that arose.
4. What did you think of the group sessions that focused on managing your lifestyle and reducing the risk factors of developing hypertension? Did you learn anything new from those sessions?

    Explore concepts which the group found useful.
5. Have you used the participant handbook since the program finished??
    a. Did the participant handbook help you in understanding high blood pressure and ways to manage it?

    Explore concept of high blood pressure
6. Have you sought help from other group members in achieving your goals and better managing your high blood pressure since the program finished?
    a. If so, what sort of support have you sought, and how did this help?
7. Have you sought help from other family members or friends in achieving your goals since the program finished?
    a. If so, what sort of support have you sought, and how did this help?
8. Have you sought help from the ASHA in your community in achieving your goals since the program finished?
    a. If so, what sort of support have you sought, and how did this help?

    Explore perceptions about the role of the ASHA from the community members
9. After setting the goals, what positive changes are you continuing?
    a. What support are you getting to help you maintain these positive changes?
    b. If no positive changes are being maintained, what are the barriers to maintaining these goals?
10. Are you managing to have your blood pressure monitored since attending the CHIRI meetings?
    a. If so, where are you going to get your blood pressure monitored?
    b. If not, could you tell me what prevents you from seeking care for your high blood pressure?
11. Could you explain where do you get your blood pressure medications?

a. If you are not obtaining medications for hypertension, why do you think you are not able to take medications regularly?

12. Is there anything else you want to tell me about the meetings that you attended, and what changes you have made since these meetings?
13. Is there anything else you want to tell me?

Appendix 2: Focus Group Discussion/In Depth Interview Guide 1b

Hypertensives Focus Group Discussion/In Depth Interview Guide – for those who did NOT attend

Questions:

1. We recently had a program to help people with high blood pressure manage their condition. Were you informed about the sessions? Could you tell me what prevented you from attending these meetings?
    a. What other barriers were there in attending these meetings?
2. What could we have done to make it easier for you to attend?
3. What type of service would you like to help you manage your high blood pressure?
4. What would have prompted you to attend these sessions?
5. Were you aware that there are changes in your lives that you could make to help manage high blood pressure?
6. Is there anything else you want to tell about these meetings on high blood pressure treatment and control?
7. The ASHAs in your village have been provided training about helping you with your high blood pressure. Are there any barriers that you would have in asking them about help to manage your high blood pressure?

    Explore the perceptions of the community with regards to the role of the ASHA.
8. Is there anything else you want to tell me?

Supplementary Table 1: Common themes from focus group discussions

Coding	Main themes	Participant experiences
Motivation to attend meeting	Information about BP and other methods to control BP	*"Doctors will tell only to reduce salt and oil. I came to know that other methods to control BP will be told here, so I decided to come." - a participant from Kerala village 2*<br>*"In general, if we go to the hospital, no doctor would check the BP and tell us about the way we should behave and exercise in any manner sir. They would write a prescription and tell us to have the medicines. But no doctor would tell us these dietary conditions like you. You have to tell!" - a participant from West Godavari village 2*
Enablers to attend	Transport to meeting<br>Close location/easy to access	*"If we were not able to come because of hot sun, they used to bring on the vehicle" - a participant from West Godavari village 3*
Reason for not attending meetings	Work<br>Away<br>Unwell<br>Bad weather	*"Sir you are asking to attend each and every meeting but it is difficult in rural areas sir. Why because we have agricultural works" - a participant from West Godavari village 6*

	Family reasons (death, celebrations)	
Barriers to attending meetings	Location of meeting<br>No medicines provided	*"If you supplied medicines also in the meetings many more would have come"* - a participant from West Godavari village 3
Information gained at meetings	Exercise<br>Dietary<br>Medication adherence	*"They are telling us to get down from an auto far from our house instead of exactly reaching our doorstep"* - a participant from West Godavari village 4<br>*"They are even showing the bowl to tell us how much quantity of curry to be taken"* - a participant from West Godavari village 4<br>*"We came to know about what we did not know earlier sir. We came to know that we should cut down pickles, oily foods, and eat more of leafy vegetables, do more exercises"* - a participant from West Godavari village 5<br>*"By attending these meetings we came to know that we have to cut down salt, oils and have to take food limitedly."* - a participant from West Godavari village 6<br>*"Now that we came to know that BP will rise if we take more salt, we have reduced the consumption of oils, non-vegetarian food and fatty food. All these illiterate people also*

		are now aware of all these things. This is the advantage of the meetings.” - a participant from West Godavari village 6<br>*“Now because of these meetings they came to know that it is compulsory to take the tablets on a regular basis, otherwise there can be paralysis attack, heart attack or else brain problems. So, they decided not to stop taking the tablets. People have become aware of the side effects of BP through these meetings.”* - a participant from West Godavari village 6
Views of meeting resources received by participants	Program resources particularly exercise sheets, portion size, weekly monitoring chart utilized post intervention	*“We came to know through these pictures what to eat and what not to eat”* - a participant from West Godavari village 6<br><br>*“Since I cannot walk, I do exercises that can be done while siting by looking into the handbook.”* - a participant from Trivandrum village 2<br><br>*“When we go to field and see them, they say that that they use these booklets.”* – ASHA Trivandrum

Support from other group members	Can be limited if group members do not live close to each other	*"All the ladies discuss about the meetings. They say to each other that if salt, chilies are taken BP will rise. Today meeting has been held etc."* - a participant from West Godavari village 6
Support from family/friends	Participants were supported by family and friends particularly in respect to dietary and physical activity lifestyle changes	*"All our household members reduced their intake of salt because of us."* - a participant from West Godavari village 4<br>*"How would we reach our goals without their cooperation sir"* - a participant from West Godavari village 5
Support from ASHAs	ASHA provided ongoing support as members of the community.	*"When we meet each other, we talk about it (BP). Will ask whether they go to check BP? Will ask whether there is any reduction (in BP)?"* - a participant from Trivandrum village 2
Ongoing goals	Simple goals such as exercise and some salt reduction goals are being maintained but difficult to	*"My BP is under control now without taking tablets because I took it as my goal. It is a big achievement."* - a participant from Trivandrum village 2

	maintain if there is no-one to monitor/review goals	*"It was useful to a timetable to put a tick mark if or not we have done that or not. Some people, even though they follow all these, they are lazy to fill the details in the form."* - a participant from West Godavari village 2
Ongoing BP monitoring	Meetings provided easy monitoring of BP, monitoring after the program cessation only occurred when attending the hospital or RMP if there is a health problem	*"Since then if there is any problem, we go the RMP doctor and get our BP checked. Otherwise generally we do not check BP"* - a participant from West Godavari village 4<br>*"Now as far as we are concerned, to get the BP checked there is no doctor in the village. If it is inevitable, we arrange someone at home and go for treatment. That is it sir, we go for checkup when we feel weakness and if there is something difference in our health"* - a participant from West Godavari village 5
Buying medicines	Purchase of medications seems to be problematic, with respect to expense, purchasing/sourcing options.	*"BP tablets given by these 104 people and my tablets are different sir. My tablets are not available here."* - a participant from West Godavari village 4<br>*"You are not supplying the BP tablets, we have to buy them from private sources, they charge 200 rupees for 15 days"* - a participant from West Godavari village 5

	Both participants and ASHAs expressed provision of medication during the meetings would be beneficial (for participation in ongoing program, and to support enhanced medication adherence)	
